# Changes in the body posture of women occurring with age

**DOI:** 10.1186/1471-2318-13-108

**Published:** 2013-10-12

**Authors:** Justyna Drzał-Grabiec, Sławomir Snela, Justyna Rykała, Justyna Podgórska, Agnieszka Banaś

**Affiliations:** 1 Institute of Physiotherapy, University of Rzeszów, Rzeszów, Poland; 2 Department of Paediatric Orthopaedics and Traumatology, Regional Hospital No 2, Rzeszow, Poland; 3 Laboratory of Molecular Biology, Institute of Obstetrics and Medical Rescue, University of Rzeszow, Rzeszow, Poland

**Keywords:** Spine, Photogrammetric method, Women

## Abstract

**Background:**

A current topic in the field of geriatrics still needing a great deal of study is the changes in body posture occurring with age. Symptoms of these changes can be observed starting between the ages of 40–50 years with a slow progression that increases after 60 years of age. The aims of this study were to evaluate parameters characterizing the posture of women over the age of 60 years compared with a control group and to determine the dynamics of body posture changes in the following decades.

**Methods:**

The study included 260 randomly selected women. The study group consisted of 130 women between the ages of 60–90 years (Older Women). The control group (Younger Women) consisted of 130 women between the ages of 20–25 years (posture stabilization period). The photogrammetric method was used to evaluate body posture using the phenomenon of the projection chamber. The study was conducted according to generally accepted principles.

**Results:**

In the analysis of parameters characterizing individual slope curves, results were varied among different age groups. The lumbar spine slope did not show significant differences between different age groups (p = 0.6952), while statistically significant differences (p = 0.0000) were found in the thoracic-lumbar spine slope (p = 0.0033) and upper thoracic spine slope. Body angle was shown to increase with age (p = 0.0000). Thoracic kyphosis depth significantly deepened with age (p = 0.0002), however, the thoracic kyphosis angle decreased with age (p = 0.0000). An increase in asymmetries was noticed, provided by a significantly higher angle of the shoulder line (p = 0.0199) and the difference in height of the lower shoulder blade angle (p = 0.0007) measurements in the group of older women.

**Conclusions:**

Changes in the parameters describing body posture throughout consecutive decades were observed. Therapy for women over the age of 60 years should involve strengthening of the erector spinae muscles and controlling body posture with the aim of reducing trunk inclination and deepening of thoracic kyphosis. Moreover, exercises shaping lumbar lordosis should be performed to prevent its flattening.

## Background

According to the World Health Organization, the geriatric population will reach 2 billion in 2050. This increase will undoubtedly enhance scientific research in the field of geriatrics.

A current topic in the field of geriatrics still needing a great deal of study is the changes in body posture occurring with age. Symptoms of these changes can be observed starting between the ages of 40–50 years with a slow progression that increases after 60 years of age. The process of changes in body posture is very complex. As a result of aging, we can observe: a decrease in the efficiency of central and peripheral neurons; a decrease in skeletal mass and muscle tissue; and weight loss. In addition, water and potassium levels within the cells are lower and the protein biosynthesis rate in muscles progressively decreases. This gradual increase in the fragility of connective tissue and the reduction of muscle strength directly affect body posture. Body mechanics are further diminished due to regressive changes in ligaments and articular cartilage. As a result of diminishing muscle strength, elderly people subconsciously try to balance themselves with supportive tools. This factor leads to further impairment of the physiological curvature of the spine, leading to compensative banding of the legs in the hip and knee joints while standing [1-3]. Describing the specific changes of body posture in the elderly population would allow for the development of targeted rehabilitation programs.

The aims of this study were to evaluate the parameters that characterize the posture of women over 60 years of age compared with a younger control group and to determine the dynamics of changes in body posture throughout consecutive decades.

## Methods

The study included 260 randomly selected women. The study group consisted of 130 women between the ages of 60–90 years (Older Women) and the control group (Younger Women) consisted of 130 women between the ages of 20–25 years (posture stabilization period). Group I was divided into three subgroups to compare body posture parameters throughout consecutive decades. All patients were ambulatory and able to remain in a standing position for tests.

The mean body height in group I was 160.5 cm ± 3.2 cm, and in group II, 165.0 cm ± 4.3 cm. The mean body weight in group I was 74.9 kg ± 11.7 kg, and in group II, 60.0 kg ± 7.6 kg. The mean BMI was 29.1 in group I and 21.4 in group II.

The primary study group included women over 60 years old who lived in the Rzeszów district, responded to the study invitation, and agreed to the proposed tests. The measurements were performed in 581 individuals. Patients with neurological disorders, mobility disorders, and those unable to hold their balance in a standing position or with the aid of orthopedic equipment (crutches, a walker, a wheelchair) were excluded from the study to maintain measurement reliability. Exclusions occurred only at the measurement stage, as the information was verified on the basis of an interview and observations. Questionable results and measurements with technical errors were also excluded from the analysis. After excluding incorrect results and individuals not meeting the inclusion criteria, a selection process without a repetition option using Statistica software was performed to randomly select 130 patients whose data were analyzed. The control group included 130 young women selected in the same manner.

**Figure 1:**
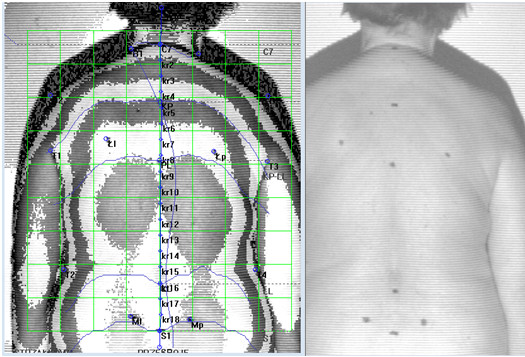
**Photogrammetric survey sample.** Source: own study. The authors obtained the patient’s consent to publish the image.

The study was conducted with the approval of the Bioethics Committee of the Medical Faculty of the University of Rzeszów. The photogrammetric method was used to evaluate body posture using the phenomenon of the projection chamber. The tests involved anthropometric measurements based on images of the studied surface. The patient is positioned at a distance of 2.6 meters from the camera while the device projects lines of strictly defined parameters on the patient’s back, allowing a spatial image to be obtained. These lines reach the patient’s back at a specific angle and are distorted depending on the distance of a given point from the device. The computer records line image distortions and numerical algorithms are used to convert them into a contour map of the surface. In optics, the physical basis of this method is called the Moire phenomenon. Thus testing is depicted in Figure 1.

Practical implementation of this method involves converting a CCD camera image into a digital signal and sending it to the computer’s memory by means of a special card. Analysis, display, and printout of the test are performed using a computer program that allows transmission of the data to the statistical software. The equipment and software used was from CQ Elektronik Systems. We decided to use the photogrammetric method due to its non-invasive, reproducible, and sensitive testing characteristics.

Photogrammetric accuracy is estimated to be 94%, while errors in radiograph assessment during Cobb angle determination were estimated to be 5,0 - 7,2% [4]. The photogrammetric method has frequently been compared with radiograph imaging by researchers because of its non-invasiveness and reproducibility. The majority of studies have compared the size of the Cobb angle in the frontal plane, while some have assessed the size of anterior-posterior curvatures.

Saad et al. compared the results of Cobb angle measurements obtained by the photogrammetric method and conventional radiograph imaging. While evaluating the reliability and accuracy of the results, the authors concluded that the Moire method, despite its high reproducibility, cannot replace conventional radiography. Nevertheless, it is useful in confirming the validity of therapies, which reduces the number of radiographs performed throughout treatment and, consequently, reduces exposure to x-ray radiation [5].

In the further research of Saad et al. on the reliability of the photogrammetric method in evaluating structural scoliosis, the authors found a strong agreement between the evaluators’ and the test-retest analyses. These results were impacted by the Cobb angle values, and in cases of higher values, a higher external agreement of the assessments was observed. The studies of Saad et al. comparing the Cobb angle values obtained from radiograph and photogrammetric testing manifested a certain agreement of results. Further confirmation of the agreement between photogrammetric testing and radiograph examination was obtained by the same team of authors, comparing thoracic kyphosis and lumbar lordosis angles in both of these examinations, in which it was also demonstrated that the results were in agreement with each other [6]. Similar studies were conducted by Leroux and Zabijek, who compared the measurements of thoracic kyphosis and lumbar lordosis in 124 patients using the radiological and photogrammetric methods; the results obtained indicated a high correlation [7]. Van Maanen et al. [8], Iunes et al. [9], and Braun and Amundson [10] confirmed the accuracy of the photogrammetry as a method of assessing posture.

This study was conducted according to generally accepted principles provided by the manufacturer [11,12]. Written, informed consent for participation in the study was obtained from participants.

The parameters measured in the study were:

KNT - angle of trunk declination; determines the vertical decline of the C7-S1 line in the frontal plane (right, left)

KPT - angle of body inclination; specifies the forward and backward incline of the body

UL - difference in height of the lower shoulder blade angles

UB - difference in depth of the lower shoulder blade angles

OL - difference in distance of the lower shoulder blade angles from the spine

KLB - angle of the shoulder line

ALPHA - slope of the lumbar spine

BETA - slope of the thoracic-lumbar spine

GAMMA - slope of the upper thoracic spine

**Figure 2:**
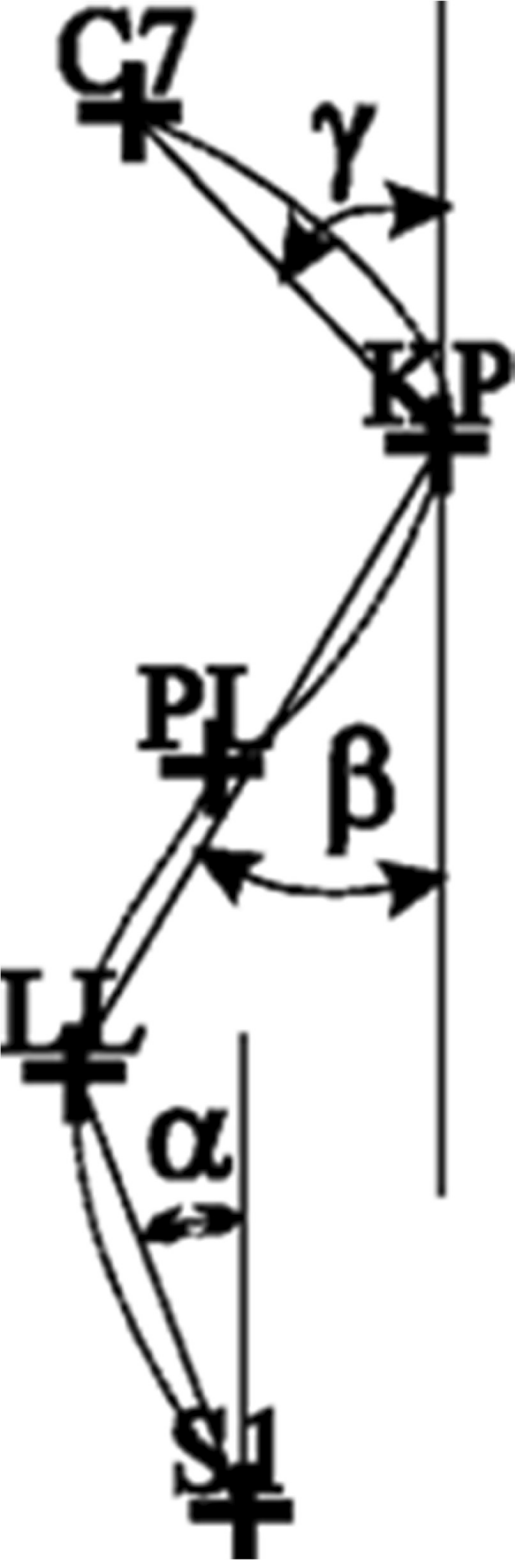
C7 - spinous process of the seventh cervical vertebra, KP - thoracic kyphosis apex, PL - transition of kyphosis into lordosis, LL - lumbar lordosis apex, S1 - transition of lumbar lordosis into the sacrum.

KLL - angle of lumbar lordosis [KLL = 180-(ALPHA + BETA) (Figure 2)]

GLL - depth of lumbar lordosis

KKP - angle of thoracic kyphosis [KKP =180-(BETA + GAMMA) (Figure 2)]

GKP - depth of thoracic kyphosis

UK - maximum deviation of spinous processes from the C7-S1 line

Angle values are reported in degrees and asymmetry values in millimeters.

In this study, the basic descriptive statistics applied for all tested parameters were mean [*x*], standard deviation [*s*], and median [Me]. Due to the lack of conformity of most distributions to the normal distribution (verified using the Shapiro-Wilk test), the non-parametric Mann–Whitney U test was used to compare parameters between groups. The test described differences between two parameters of the two groups, with a statistical significance of p < 0.05. To compare the results between the three age groups, the Kruskal-Wallis ANOVA test was applied. Due to the lack of conformity of most distributions to the normal distribution (verified by the Shapiro-Wilk test), a non-parametric test was used. The statistical significance level was p < 0.05. When significant differences arose between the mean values of the groups, verified by the Kruskal-Wallis ANOVA test, the multiple comparison test was used, representing detailed relationships between groups.

## Results

The results are presented in tabular form in Tables 1 and 2 and statistically significant data is highlighted in red. Comparing the parameters of body posture in women over the age of 60 with the control group, statistically significant differences occurred between most of the tested parameters. Analyzing parameters of the individual slope curves, results varied among age groups and the ALPHA angle was not significantly different between groups (p = 0.6952). Concerning the BETA angle (p = 0.0033) and the GAMMA angle, a statistical difference was found (p = 0.0000). Additionally, the body angle showed an increase with age (p = 0.0000). Thoracic kyphosis was significantly deepened with age (p = 0.0002), however, the thoracic kyphosis angle decreased with age (p = 0.0000). An increase in asymmetries was also noticed, provided by significantly higher KLB (p = 0.0199) and UL (p = 0.0007) measurements in the group of older women (Table 1).

**Table 1:** Comparison of parameters characterizing body posture in the study group and the control group

**Variable**	**Older women**			**Younger women**			**MANN–WHITNEY U**	
***xx***	***x***	**Me**	***s***	***x***	**Me**	***s***	**Z**	**p**
ALPHA [°]	24.53	14.10	25.67	26.74	14.50	24.96	0.3919	0.6952
BETA [°]	5.53	4.50	4.23	4.08	3.00	4.72	-2.9358	0.0033
GAMMA [°]	41.25	37.80	23.25	25.92	14.05	21.49	-4.9632	0.0000
KPT [°]	-11.88	-9.70	17.13	-2.03	-3.55	14.92	4.3808	0.0000
KKP [°]	138.31	147.20	21.76	150.55	161.45	21.29	4.3320	0.0000
GKP [mm]	-7.02	-7.50	19.90	3.60	3.80	9.55	3.7103	0.0002
KLL [°]	185.86	170.70	34.09	186.68	167.45	35.72	-1.6231	0.1046
GLL [mm]	-6.15	-8.30	14.19	-12.74	-9.80	28.04	-1.5296	0.1261
KNT [°]	0.13	0.10	1.43	-0.65	-0.55	1.24	-3.6393	0.0003
KLB [mm]	2.52	0.00	9.84	-0.88	0.00	8.70	-2.3285	0.0199
UL [mm]	2.38	1.90	12.32	-1.28	-0.90	7.05	-3.3831	0.0007
UB [mm]	-0.65	-0.80	8.52	-1.64	-0.80	7.13	-0.7035	0.4818
OL [mm]	-3.67	-3.80	12.50	-0.80	-0.05	12.40	1.5676	0.1170
UK [mm]	-0.60	-1.40	6.06	-2.00	-2.90	4.88	-1.6231	0.1046

The statistical significance level was p < 0.05.

**Table 2:** Dynamics of the changes in body posture parameters in the study group

**Variable**	**Age group: 60–70 years n = 50**			**Age group: 71–80 years n = 45**			**Age group: 81–90 years n = 35**			**Kruskal-Wallis ANOVA**
***x***	***x***	**Me**	***s***	***x***	**Me**	***s***	***x***	**Me**	***s***	**p**
ALPHA [°]	25.18	13.50	25.83	22.91	14.50	25.39	25.28	14.10	27.48	0.9999
BETA [°]	5.36	5.20	3.35	4.63	3.40	4.01	8.32	6.30	6.76	0.1436
GAMMA [°]	38.42	47.30	22.66	40.97	30.40	24.19	54.28	68.10	20.74	0.0204
KPT [°]	-10.68	-8.00	15.74	-11.55	-9.70	19.25	-17.89	-16.20	18.12	0.1708
KKP [°]	138.82	125.70	22.27	139.39	152.20	22.57	133.68	126.70	18.34	0.5192
GKP [mm]	-0.47	2.30	16.34	-10.88	-12.80	19.28	-27.16	-27.10	21.02	0.0001
KLL [°]	184.44	168.60	34.43	183.88	170.70	33.59	196.51	180.00	34.41	0.0040
GLL [mm]	-8.93	-10.50	11.14	-7.09	-7.50	12.97	8.15	9.00	20.29	0.0075
KNT [°]	-0.02	-0.30	1.16	0.49	0.40	1.21	-0.08	0.60	2.57	0.2424
KLB [mm]	1.81	0.00	9.04	4.47	0.90	11.35	1.28	1.90	9.77	0.6050
UL [mm]	2.82	1.90	8.22	5.22	9.50	12.69	-5.82	-3.80	21.34	0.0318
UB [mm]	-0.72	0.00	7.48	-0.85	-3.00	9.73	0.12	0.00	10.47	0.8803
OL [mm]	-4.65	-4.80	11.20	-0.99	0.10	11.89	-5.37	-5.70	18.32	0.3376
UK [mm]	-0.17	1.00	5.63	-1.73	-4.40	6.19	0.05	-1.90	7.65	0.3656

The statistical significance level was p < 0.05.

Analyzing the dynamics of parameters characterizing body posture throughout consecutive decades, several important trends were observed. There was a statistically significant difference between the GAMMA angle in the group of women aged 60–70 years and 81–90 years (p = 0.0168) and the GAMMA angle increased with age. There was also a statistically significant difference between the GKP parameter in women of 81–90 years and 60–70 years (p = 0.0001) and among women of 71–80 years and 60–70 years (p = 0.0467). However, there was no significant difference in the GKP parameter between the groups of women between the ages of 71–80 years and 81–90 years (p = 0.0945). The mean value of GKP decreased with age. The KLL parameter differentiates essentially between the age groups of 60–70 years and 81–90 years (p = 0.0027) and between the age groups of 81–90 years and 71–80 years (p = 0.0382). Thus, there was no significant difference in the KLL parameter between the age groups of 60–70 years and 71–80 years (p = 1.0000), while the mean value of the KLL parameter increased with age. In addition, there was a statistically significant difference in the GLL parameter between the age groups of 81–90 years and 60–70 years (p = 0.0053), while the average value of the GLL parameter increased with age. The UL parameter significantly differed between the groups of women between 71–80 years of age and 81–90 years of age (p = 0.0263) (Table 2).

## Discussion

Our findings show that significant changes were observed in the body posture of women over 60 years of age in comparison with the control group. In the study group, the thoracic kyphosis angle is increased when compared to that of the control group. The parameters characterizing the dimension of lumbar lordosis were not changed, nor were they in the UB, OL, or UK parameters. The other parameter measurements are significantly higher in older women. Observing the changes in the parameters describing body posture throughout consecutive decades, differences were found among age groups in the upper thoracic spine slope, thoracic kyphosis depth, lumbar lordosis angle, and asymmetry of the shoulder blades. Other parameters did not show regular or significant trends.

In the studies of Anwajler et al., thoracic kyphosis depth in women is significantly increased [13]. Singh et al. compares the shape of the anterior-posterior curvature of the spine in a group of younger and older women, where the authors found significant deepening of thoracic kyphosis in older women. In the case of lumbar lordosis, the same authors reported no significant differences [14]. These results are confirmed by the research and results of Kado [15]. A great number of studies is devoted to the relationship between thoracic kyphosis and increasing age [16-18]. In our study, thoracic kyphosis depth increased gradually with age in groups of women aged 60–70 years, 71–80 years, and 81–90 years. Similar results were obtained by other authors [19-22]. The cause of the deepening of thoracic kyphosis with age is multifactorial; the aging process causes changes in the body in an upright position due to changes in passive and active stabilizers of the spine [23-26]. This contributes to the development of degenerative-deforming processes, especially at the spine and hip joints, which are particularly vulnerable to weight load. Regressive changes in ligaments and articular cartilage cause deterioration of body mechanics, progressing with age. As a result of diminishing muscle strength, elderly people subconsciously balance their body weight by adjusting the spine, which significantly affects body posture. This leads to further impairment of the physiological curvature of the spine, and when in the standing position, to compensative banding of the legs in the hip and knee joints. Tilting of the whole body forward results in movement of the center of gravity forward in the same direction [13]. As previously reported, increased thoracic kyphosis results in the progression of disability [27], an increase in falls due to the transfer of the center of gravity [27-29], lung disease [30], diminished quality of life [21], an increased risk of fractures [31], and overload disease [32]. Therefore, the deepening of kyphosis with age in women, who face this problem more frequently than men, receives much attention in current literature [20,21]. According to Hammerberg et al. and Gelb et al., there is a correlation between age and reduced lumbar lordosis [33,34]. The additional parameters characterizing body posture that were analyzed in our study have not yet been described by other authors, hence, there is a lack of opportunity to compare our results with those of other authors.

The obtained results are clinically important as they concern parameters significantly affecting the quality of life in patients over the age of 60 years. Deepening of thoracic kyphosis, flattening of lumbar lordosis, and asymmetries result in back pain syndrome. Exacerbation of these pathologies in the consecutive decades of life requires physiotherapy in geriatric patients to prevent or postpone involutional changes of the spine. Targeted preventive physiotherapy will significantly improve the fitness and health of the population over 60 years of age.

Our findings constitute the first analysis in such detail of body posture parameters and can be translated into clinical practice, as well as individual, targeted rehabilitation schedules for elderly patients. Only targeted therapy allows for achievement of the expected results, and the outcomes reported in this paper indicate that older and younger women differ significantly in body posture, making the application of universal instructions and exercises in patients of all ages impossible. Normal values for lumbar lordosis and thoracic kyphosis are different in younger and older women, and also different for women over 60 years of age and throughout consecutive decades.

Due to the negative impact of thoracic kyphosis on the quality of life, it is an important parameter to correct. However, it is crucial not to ignore other parameters characterizing body posture, as each therapy should be comprehensive and cause-oriented. Dealing merely with the thoracic area, only change a small part of the biomechanical system of the spine can be changed. Our research shows significant differences in the body angle, the slope of various curves of the spine, and trunk asymmetry with age. All of these changes in the spine should be considered in the therapy of older patients. Both the change in the slope of the whole body, as well as changes in the slope of individual sections of the spine, will affect abnormal load balance and will change the parameters of body posture, which may contribute to an increase in the frequency of falls in the elderly [35,36]. Asymmetries in the spine are permanent changes in adults and, if not prevented, may deepen, which can cause pain. Changes in the body posture of women after the age of 60 years must be taken into account in collaboration with geriatric patients. Due to the fact that non-invasive methods of posture and spinal assessment are currently available, each patient should be examined prior to therapy and an exercise plan should be adapted individually, concurrent with therapy, to effectively monitor its effects.

Despite being the first analysis in such detail of body posture parameters, this study has some limitations; our evaluation did not involve muscle strength measurements or back pain level assessment.

## Conclusions

Observing the changes in the parameters describing body posture throughout consecutive decades differences were found among age groups in the slope of the upper thoracic spine, the depth of thoracic kyphosis, the angle of lumbar lordosis, and asymmetry of the shoulder blades.

Therapy for women over the age of 60 years should involve strengthening of the erector spinae muscles and controlling body posture with the aim of reducing trunk inclination and deepening of thoracic kyphosis. Moreover, exercises shaping lumbar lordosis should be performed to prevent its flattening.

## Competing interests

The authors declare that they have no competing interests.

## Authors’ contributions

JD-G designed data collection tools, monitored data collection for the entire trial, wrote the statistical analysis plan, organized and analyzed the data, and drafted and revised the paper. She is the guarantor. SS designed data collection tools, monitored data collection for the entire trial, and revised the draft paper. JR analyzed the data and drafted and revised the paper. JP analyzed the data and drafted and revised the paper. AB initiated the collaborative project and drafted and revised the paper. All authors read and approved the final manuscript.

## Pre-publication history

The pre-publication history for this paper can be accessed here:

http://www.biomedcentral.com/1471-2318/13/108/prepub
